# Lighting and perceptual cues: Effects on gait measures of older adults at high and low risk for falls

**DOI:** 10.1186/1471-2318-11-49

**Published:** 2011-08-24

**Authors:** Mariana G Figueiro, Barbara Plitnick, Mary S Rea, Laura Z Gras, Mark S Rea

**Affiliations:** 1 Lighting Research Center, Rensselaer Polytechnic Institute, 21 Union Street, Troy, NY, 12180, USA; 2 The Sage Colleges, 65 FirstStreet, Troy, NY, 12180, USA

**Keywords:** Lighting, perceptual cues, and falls risk

## Abstract

**Background:**

The visual system plays an important role in maintaining balance. As a person ages, gait becomes slower and stride becomes shorter, especially in dimly lighted environments. Falls risk has been associated with reduced speed and increased gait variability.

**Methods:**

Twenty-four older adults (half identified at risk for falls) experienced three lighting conditions: pathway illuminated by 1) general ceiling-mounted fixtures, 2) conventional plug-in night lights and 3) plug-in night lights supplemented by laser lines outlining the pathway. Gait measures were collected using the GAITRite^© ^walkway system.

**Results:**

Participants performed best under the general ceiling-mounted light system and worst under the night light alone. The pathway plus night lights increased gait velocity and reduced step length variability compared to the night lights alone in those at greater risk of falling.

**Conclusions:**

Practically, when navigating in more challenging environments, such as in low-level ambient illumination, the addition of perceptual cues that define the horizontal walking plane can potentially reduce falls risks in older adults.

## Background

Risk for falls increases with age and poses major threats to the independence of older adults living at home and in more controlled environments. The visual system plays an important role in maintaining balance while moving in and around the environment [1-3]. Persons with severe deficits in the vestibular or the somatosensory systems rely heavily on visual cues; they will lose balance if visual information is removed by eyes closure [4]. The dependence on visual information for maintenance of postural stability and control increases with age due to age-related changes that occur in the vestibular and somatosensory systems [5-7]. In fact, older adults with impaired vision show decreased walking speed, a shift in the center of mass over the center of the base of support, and an increased variability in center of mass upon termination of walking [8,9]. Even seemingly small reductions in visibility affect balance. Older adults with high levels of gait disorder exhibit more variable and less steady gaits when walking in dim lighting conditions [10]. Even for healthy older adults, although to a lesser extent, gait becomes slower and step lengths shorter in dim lighting conditions [10]. Age-related changes to the vestibular and the somatosensory systems, *together with *age-related changes to the visual system result in severely impaired balance control, leading to the increased risk for falls commonly found among older adults [1]. Because lighting affects one's ability to acquire visual information about the environment and because age-related changes in the visual system compromise the acquisition of that information, novel lighting systems that enhance visual perception of the environment could play a very important role in maintaining balance in older adults with and without gait disorders.

Figueiro et al. [11] tested balance control in healthy older adults who were at low risk for falls, while viewing a novel luminous doorframe. They used the sit-to-stand (STS) test to collect balance control data from twelve older adults when the test-room was dimly illuminated by conventional nightlights or by the luminous doorframe. The luminous doorframe could be tilted left or right or could be oriented to provide veridical horizontal and vertical (H/V) cues about the environment. The luminous doorframe that provided H/V visual cues significantly reduced sway in the early phase of the STS task relative to the conventional nightlights. Moreover, posture while rising was directly influenced by the tilt angle; tilting it to the right or to the left caused subjects to lean to the right or to the left, respectively, while rising.

Because of poorer balance control, older adults tend to look down when walking in more challenging environments [12]. For this situation, a luminous doorframe will have limited utility. The present study was designed to extend the results by Figueiro et al. [11] by investigating the impact of a second novel lighting system based upon the same idea that providing enhanced veridical perceptual cues to older adults should improve their balance control. It was hypothesized that, together with low-level illumination from conventional nightlights, laser lines that demarcate a pathway should provide older adults with perceptual cues about the horizontal walking plane, and therefore, improve gait measures in two populations of older adults, those with high and low falls risks (HFR and LFR, respectively). Furthermore, it was hypothesized that this improvement would be greater for those at HFR.

The effectiveness of a second novel lighting solution designed to provide low-level ambient illumination together with enhanced horizontal plane information to a person while walking was tested using the GAITRite^© ^Mat (CIR systems, Havertown, PA). Although the GAITRite machine reports a variety of outcome measures, only a) Step Length, b) Stride Length Difference, and c) Velocity measures were examined because these have been the measures most associated with risks for falls. From these measures the d) standard deviation (SD) of the Step Length, the e) SD of the Stride Length Difference, and the f) SD of Velocity were calculated, because, again, these gait variability measures have been associated with falls risk. Therefore, these measures were selected as the most important for comparing the different lighting conditions in terms of falls risk [13,14].

## Methods

### Subject Selection

Twenty-four adults age 65 years or older were recruited to participate in the study. Subjects were recruited through retirement communities, assisted living facilities, and older adult centers in the Albany, New York area. Subjects were paid for their participation in the study. The Institute Review Boards (IRB) of both Rensselaer Polytechnic Institute and The Sage Colleges approved the study, and informed consent was obtained from every participant.

Of those recruited and accepted to the study, 12 had a history of falls within the past six months, and 12 had not fallen during this period. Demographic characteristics for all participants are reported in Table 1. Falls risk was assessed using the Berg Balance Scale [15], which is a 14-item scale designed to measure balance of older adults in a clinical setting. Subjects were asked to complete 14 tasks determined to be representative of daily activities that require balance (e.g., sitting to standing, retrieving objects from floor, standing with eyes closed, standing on one foot, turning to look behind) and each task is rated by an examiner on a 5-point scale ranging from zero (cannot perform) to 4 (normal performance). Some tasks are rated according to the quality of performance while others are rated according to the time taken to complete the task. Overall scores range from zero (severely impaired balance) to 56 (excellent balance). Those subjects who scored 45 or lower on this scale and who had reported to have fallen at least two times within the past six months were considered HFR [16]. Those who scored 46 or higher and who had not fallen in the past six months were eligible for inclusion in the LFR category.

**Table 1:** Demographic characteristics for all participants presented as mean (standard deviation)

	High Falls Risk (HFR)			Low Falls Risk (LFR)		
	All (n = 12)	Male (n = 4)	Female (n = 8)	All (n = 12)	Male (n = 4)	Female (n = 8)
**Age (yrs)**	82 (9)	80 (4)	83 (10)	75 (6)	73 (8)	75 (6)

**Height (cm)**	160 (13)	175 (8)	155 (8)	165 (8)	175 (5)	160 (5)

**Weight (kg)**	69 (1)	74 (15)	66 (19)	71 (15)	85 (11)	64 (11)

BMI*	27 (7)	24 (3)	28 (8)	25 (3)	28 (3)	24 (3)

**Blood Pressure**	135/73 (13/4)	131/71 (8/1)	138/74 (15/4)	136/74 (7/5)	134/76 (11/5)	137/73 (5/5)

**Snellen acuity**	20/30 (9)	20/25 (4)	20/33 (10)	20/27 (9)	20/23 (9)	20/29 (9)

**Berg Balance score**	43 (2)	44 (0)	42 (3)	55 (2)	55 (3)	55 (2)

* Body Mass Index

Functional status was measured at baseline using the Minimum Data Set Activities of Daily Living Scale (MDS-ADL) [17]. The MDS-ADL assigns subjects a score ranging from 0 (independent/no assistance) to 4 (total dependency) over the last seven days for the following seven items: bed mobility, eating, locomotion, transfer, toileting, dressing, and personal hygiene. The items are summed to yield a scale that ranges from 0 to 28. All subjects scored 0. All subjects self-reported being free from cataracts, macular degeneration, and glaucoma. Visual acuity was measured using a Snellen eye chart. Visual acuity was ascertained binocularly and participants were wearing their habitual optical correction when they underwent the test. All subjects had normal color vision as measured by the Ishihara test.

Prior to being accepted into the study, potential participants were interviewed about their health status. Exclusion criteria for all subjects included major organ failure, major illness, high blood pressure (greater than 140/90 as defined by the American Heart Association), stroke, history of brain injury, or uncontrolled generalized disorders such as diabetes, as well as the use of psychotropic (sleep aid) medicine. A registered nurse measured subject's blood pressure at the recruitment site and again just prior to performing the experiment.

### Lighting Conditions

**Figure 1:**
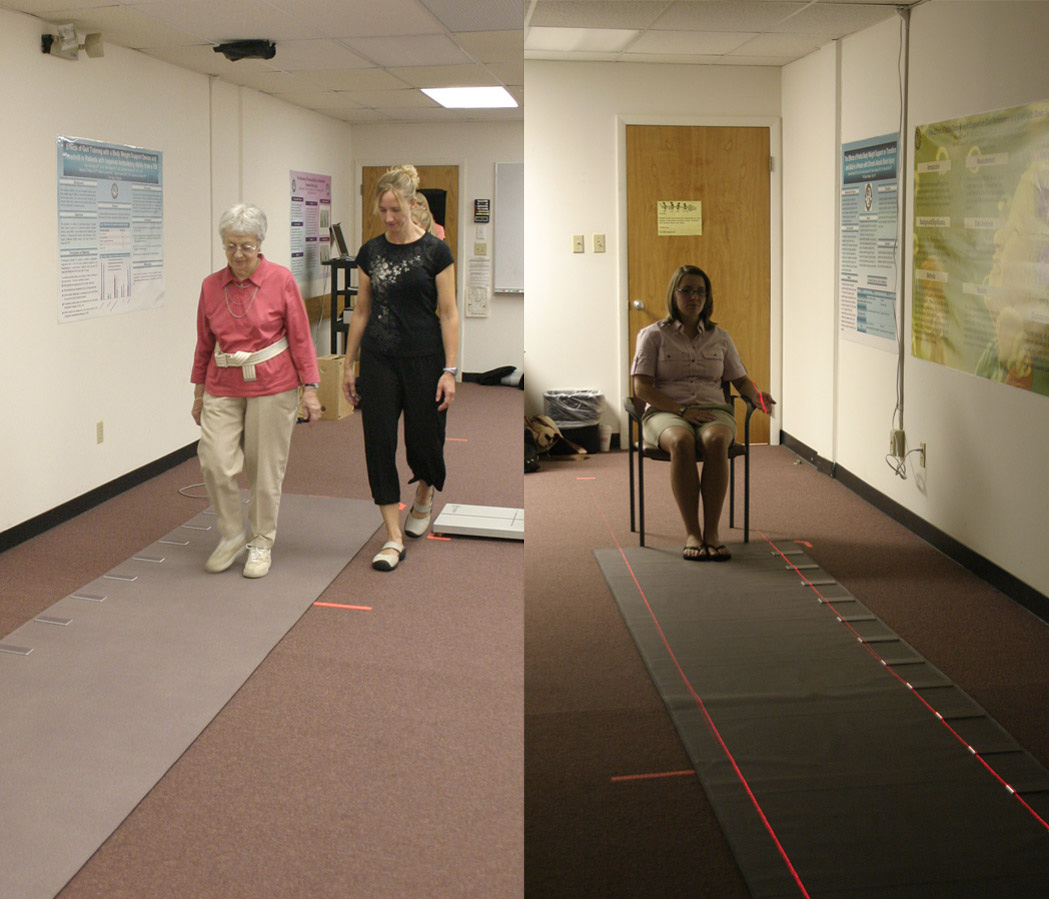
**Photograph of the pathway lighting (ambient lights were turned on for the photograph, but only night lights were on during the experiment)**.

Three lighting conditions were tested: 1) ambient illumination provided by 16 ceiling-mounted fixtures (650 lux at the cornea); 2) two conventional plug-in incandescent night lights (0.015 lux at the cornea); 3) two plug-in night lights supplemented by laser lines outlining the pathway (0.015 lux at the cornea). The ceiling-mounted fixtures were sixteen common 2' × 4' lensed fluorescent troffers. Six of them contained four and ten of them contained two F32T8 lamps. The two night lights, each containing a single 6W incandescent lamp, are typical of those used in older adults' bedrooms and bathrooms after bedtimes. Four laser leveling devices (Craftsman, Laser Trac Level, model # 48247) were suspended from the ceiling to direct a narrow pencil of 650 nm peak wavelength (red) light onto the mat to outline the pathway (Figure 1). The laser pathway lights were only used with the incandescent night lights (third lighting condition) and did not affect the illuminance level measured at the cornea.

### Procedures

The GAITRite^© ^Mat was used to measure the subject's gait and step length. The GAITRite^© ^consists of a 4.6-meter (12 feet) long flexible mat with 13,824 built-in pressure activated sensors that are arranged in a 48 × 288 grid pattern, and a sample rate of 80 Hz. These sensors record when pressure is applied to them and the information is then sent to a computer with the GAITRite^® ^program. The mat has a spatial resolution of 0.5" (1.27 cm). Temporal aspects of gait are calculated automatically by recording the amount of time between sensor activations. Subjects were instructed to begin walking two meters before the mat threshold (to ensure that they were demonstrating their steady gait pattern), walk over the mat at a comfortable speed, and continue walking two meters beyond the end of the mat. Subjects were asked to perform four trials under every lighting condition, which were presented to them in a counterbalanced manner; every subject served as his/her own control, experiencing all three lighting conditions. Subjects sat for twenty minutes prior to starting the first trial under each lighting condition to pre-adapt their eyes.

### Data Analyses

Step Length in centimeters (cm) was measured on the horizontal axis of the walkway from the heel point of the current footfall to the heel point of the previous footfall on the opposite foot. The Step Length can be a negative value if the patient fails to bring the landing foot heel point forward of the stationary foot heel point. Using Excel spreadsheets, the averages of the Step Lengths as well as the SDs between Step Lengths were determined for each trial and are reported separately. It was hypothesized those who had worse balance would exhibit greater Step Length SD. The SD of the Step Length for each trial, calculated using the Excel spreadsheet function for calculating SD, was used as a measure of gait variability. Because we hypothesized that the novel lighting solution would improve gait measures in the two populations of older adults (LFR and HFR) and that the improvement would be more for those at greater risk of falling, we planned to compare the effect of each lighting condition on each group separately (HFR and LFR) for each outcome measure, using post-hoc two-tailed Student's t-tests.

Stride length in cm was measured on the line of progression between the heel points of two consecutive footfalls of the same foot (left to left, right to right). The left and the right Stride Lengths were averaged for each subject for each trial, resulting in one average number for right and one average number for left Stride Length. The absolute difference was then calculated for each of these averaged measures by subtracting one from the other and this Stride Length Difference (left-right) was used as a dependent measure. The SD between the Stride Length Differences of the four trials was also calculated using the Excel spreadsheet function for calculating SD, and was used as a measure of gait variability. It was hypothesized those who had worse balance would exhibit greater Stride Length Difference SD.

Velocity (cm/s) was obtained after dividing the distance by the time elapsed between the first contacts of the first and the last footfalls. The SD of Velocity was also calculated using the Excel spreadsheet function for calculating SD, and was used as a measure of gait variability. It was hypothesized those who had worse balance would exhibit greater Velocity SD.

A one-between (HFR vs. LFR), two-within (three lighting conditions × three trials) mixed design analyses of variance (ANOVA) was performed for Step Length, Stride Length Difference, SD of Step Length and SD of Velocity. Because trials were used to calculate the SD of Stride Length Difference and Velocity, a one-between (HFR vs. LFR), one-within (lighting conditions) ANOVA was performed for these outcome measures. All statistical analyses were performed using PASWStatistics 18.0. Two-tailed post-hoc Student's t-tests were performed to further examine the main effects and the interactions between the experimental variables. Bonferroni corrections were made to the pairwise comparisons to limit Type 1 errors.

## Results

Table 2 provides the results of the ANOVAs for the six outcome measures examined here. Depending upon the outcome measure, the groups, the trials, and/or the lighting conditions main effects reached statistical significance. No interaction terms were statistically significant for any outcome measures; therefore, they are not reported in the table. Those outcome measures where the main effects reached statistical significance are discussed below.

**Table 2:** Statistical analyses for the six dependent measures

Variable	F value	P value
**Step Length**		

Lighting Condition	F_2,44 _= 10.95	**< .0001**

Trial	F_3,66 _= 13.10	**< .0001**

Groups (HFR v. LFR*)	F_1,22 _= 36.75	**< .0001**

**Stride Length Difference**		

Lighting Condition	F_2,44 _= 1.81	0.180

Trial	F_3,66 _= 0.78	0.489

Groups (HFR v. LFR)	F_1,22 _= 3.73	0.067

**Velocity**		

Lighting Condition	F_2,44 _= 6.56	**0.004**

Trial	F_3,66 _= 14.99	**< .0001**

Groups (HFR v. LFR)	F_1,22 _= 38.06	**< .0001**

**SD Step Length**		

Lighting Condition	F_2,42 _= 5.38	**0.010**

Trial	F_3,63 _= 0.88	0.418

Groups (HFR v. LFR)	F_1,21 _= 10.53	**0.004**

**SD Stride Length Difference**		

Lighting Condition	F_2,44 _= 4.89	**0.014**

Groups (HFR v. LFR)	F_1,22 _= 3.87	0.062

**SD Velocity**		

Lighting Condition	F_2,44 _= 1.02	0.344

Groups (HFR v. LFR)	F_1,22 _= 0.05	0.824

* High Falls Risk (HFR) v. Low Falls Risk (LFR)

### Step Length

**Figure 2:**
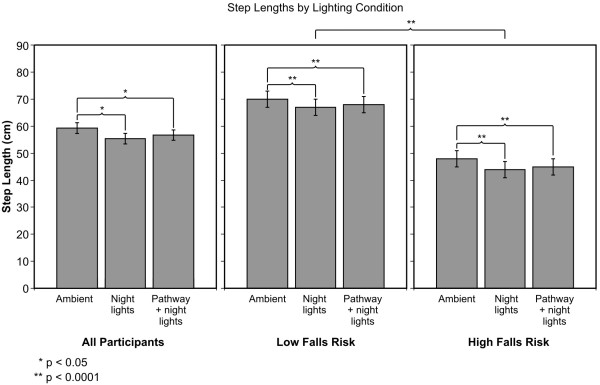
**Average ± SEM for Step Length under the three lighting conditions**. Average values for all participants are shown together with those for HFR and LFR older adults.

The ANOVA revealed a significant main effect of lighting conditions (F_2,44 _= 10.95; p < 0.0001). Figure 2 shows the average ± standard error of the mean (SEM) Step Lengths for the three lighting conditions; the average ± SEM of Step Length was 59 ± 2 cm under ambient illumination, 55 ± 1.9 cm under night lights alone, and 57 ± 1.9 cm under the pathway plus night lights condition. Post-hoc two-tailed t-tests revealed that participants had a significantly greater Step Length under ambient illumination compared to night lights alone (p = 0.001) and to pathway plus night lights (p = 0.007). The difference between night lights alone and pathway plus night lights did not reach statistical significance (p = 0.44). A significant difference (F_1,22 _= 36.75; p < 0.0001) between groups (HFR vs. LFR) was also found; the average ± SEM Step Length for those at HFR was 46 ± 3 cm and for LFR was 68 ± 3 cm.

There was also a significant main effect of trials (F_3,66 _= 13.1; p < 0.0001). The average ± SEM Step Length was 56 ± 2 cm on trial 1, 57 ± 2 cm on trial 2, 58 ± 2 cm on trial 3, and 58 ± 2 cm on trial 4. Participants had a significantly shorter Step Length on trial 1 than on trial 3 (p < 0.0001) and on trial 4 (p = 0.008). Participants also had a significantly shorter Step Length on trial 2 than on trial 3 (p < 0.0001) but this difference did not reach significance when compared to trial 4 (p = 0.025).

Although the groups by lighting condition interaction was not statistically significant, we hypothesized that older adults at HFR would be more affected by the lighting conditions than those at LFR. In order to verify our hypothesis, we performed post-hoc two-tailed paired t-tests on Step Lengths, comparing both groups at each lighting condition. For the older adults at HFR, Step Length was significantly greater under ambient illumination than under night lights alone (p < 0.0001) and under pathway plus night lights (p = 0.001), but there was no statistical difference between Step Length under night lights alone and pathway plus night lights (p = 0.049). The average ± SEM Step Length in HFR older adults was 48 ± 3 cm under ambient illumination, 44 ± 3 cm under night lights alone, and 45 ± 3 cm under pathway plus night lights. For the older adults at LFR, Step Length was significantly greater under ambient illumination than under both night lights alone (p < 0.0001) and pathway plus night lights (p < 0.0001), but there was no statistical difference between Step Length under night lights alone and pathway plus night lights (p = 0.115). The average ± SEM Step Length in LFR was 70 ± 3 cm under ambient illumination, 67 ± 3 cm under night lights alone, and 68 ± 3 cm under pathway plus night lights.

### SD of the Step Length

**Figure 3:**
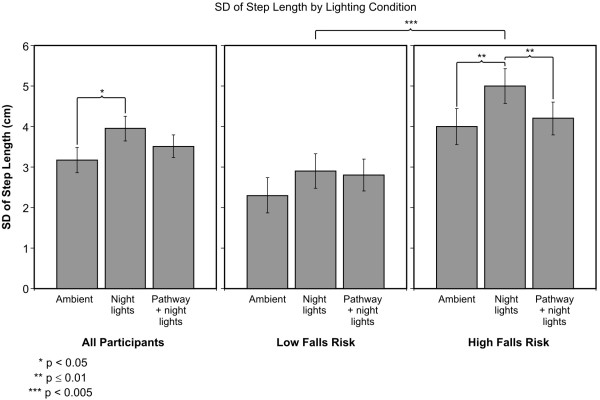
**Average ± SEM of the SD of the Step Length under all three lighting conditions**. Average values for all participants are shown together with those for HFR and LFR older adults.

Although the average number of Step Lengths was recorded, the spreadsheet obtained from the GAITRite^© ^report did not have data for the individual steps taken by one subject from the HFR group; therefore, we could not calculate SD of the Step Length for this subject. Based upon the remaining data, the ANOVA showed a significant main effect of lighting conditions (F_2,42 _= 5.38; p = 0.010). The average SD of the Step Length ± SEM was 3.2 ± 0.31 cm under ambient lights, 4.0 ± 0.30 cm under night lights alone and 3.5 ± 0.29 cm under pathway plus night lights (Figure 3). Post-hoc two-tailed t-tests revealed that participants had a significantly greater SD of the Step Length under night lights alone than under ambient illumination (p = 0.009). The SD of the Step Length under night lights alone was not statistically different than under pathway plus night lights (p = 0.057). The difference between ambient illumination and pathway plus night lights did not reach statistical significance (p = 0.139).

The ANOVA also revealed a significant difference (F_1,21 _= 10.5; p = 0.004) between groups (HFR vs. LFR); the average SD of the Step Length ± SEM for those at HFR was 4.4 ± 0.4 cm and for LFR was 2.7 ± 0.36 cm. Although the groups by lighting condition for the SD of Step Length was not significant, in order to verify our hypothesis that the pathway plus night lights would reduce SD of Step Length for HFR older adults more than for LFR, we performed post-hoc two-tailed paired t-tests on the SD of the Step Length comparing both groups at each lighting condition. HFR participants had significantly greater SD of the Step Length under night lights alone than under both ambient illumination (p = 0.01) and pathway plus night lights (p = 0.01). The SD of the Step Length was not significantly different between pathway plus night lights and ambient illumination (p = 0.36). The average ± SEM SD of the Step Length was 4.0 ± 0.45 cm under ambient illumination, 5.0 ± 0.43 cm under night lights alone, and 4.2 ± 0.40 cm under pathway plus night lights. For the LFR participants, after adjusting for multiple comparisons, the SD of the Step Length was not significantly lower under ambient illumination than under night lights alone (p = 0.021). There was also no statistically significant difference in SD of the Step Length under ambient illumination and under pathway plus night lights (p = 0.755). The SD of the Step Length under night lights alone and under pathway plus night lights was not statistically significantly different either (p = 0.076). The average ± SEM SD of the Step Length in LFR older adults was 2.3 ± 0.43 cm under ambient illumination, 2.9 ± 0.42 cm under night lights alone, and 2.8 ± 0.39 cm under pathway plus night lights.

### SD of the Stride Length Difference

**Figure 4:**
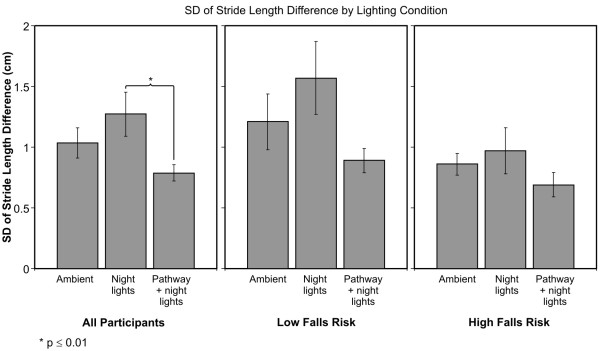
**Average ± SEM of SD of Stride Length Difference under the three lighting conditions**. Average values for all participants are shown together with those for HFR and LFR older adults.

Again, the GAITRite^© ^report did not have data for the individual steps taken by one subject who was at HFR; therefore, the SD of the Stride Length Difference could not be calculated for that subject. The ANOVA based upon the remaining data revealed only a significant main effect of lighting conditions (F_2,44 _= 4.89; p = 0.012). The average SD of the Stride Length Difference ± SEM was 1.0 ± 0.12 cm under ambient illumination, 1.3 ± 0.18 cm under night lights alone, and 0.8 ± 0.07 cm under pathway plus night lights (Figure 4). Post-hoc two-tailed t-tests revealed that participants had a significantly greater SD of the Stride Length Difference for the night lights alone than for the pathway plus night lights (p = 0.01). There was no statistically significant difference in SD Stride Length Difference between ambient illumination and night lights alone (p = 0.100), and between ambient illumination and pathway plus night lights (p = 0.114).

We also performed post-hoc two-tailed paired t-tests on the SD of the Stride Length Difference for each group at each lighting condition. None of the pairwise comparisons between lighting conditions achieved significance in older adults at HFR (p = 0.520 for ambient illumination vs. night light alone; p = 0.137 for night lights alone and pathway plus night lights; and p = 0.139 for ambient illumination and pathway plus night lights). The average ± SEM SD Stride Length Difference in HFR older adults was 0.86 ± 0.09 cm under ambient illumination, 0.97 ± 0.19 cm under night lights alone and 0.69 ± 0.10 cm under pathway plus night lights. For the LFR participants, after adjusting for multiple comparisons, the SD of the Stride Length Difference was not significantly lower under pathway plus night lights than under night lights alone (p = 0.044). There was no statistically significant difference in SD of the Stride Length Difference between ambient illumination and both night lights alone (p = 0.129), and pathway plus night lights (p = 0.274). The average ± SEM SD of the Stride Length Difference in LFR older adults was 1.21 ± 0.23 cm under ambient illumination, 1.57 ± 0.30 cm under night lights alone and 0.89 ± 0.10 cm under pathway plus night lights.

### Velocity

**Figure 5:**
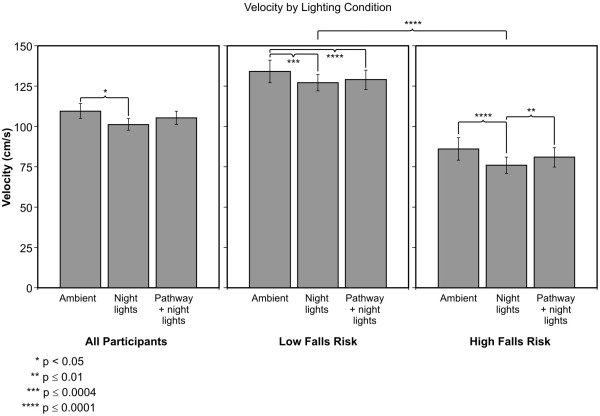
**Average ± SEM of velocity under the three lighting conditions**. Average values for all participants are shown together with those for HFR and LFR older adults.

The ANOVA revealed a significant main effect of lighting conditions (F_2,44 _= 6.56; p = 0.004). The average ± SEM Velocity in cm/s was 110 ± 5 when participants were performing the test under ambient lights, 101 ± 4 cm/s under the night lights alone and 105 ± 4 cm/s under the pathway plus night lights (Figure 5). Post-hoc two-tailed t-tests revealed that participants were significantly faster under ambient illumination than under night lights alone (p = 0.002) but not under pathway plus night lights (p = 0.052). The difference between night lights alone and pathway plus night lights did not reach significance (p = 0.102). The ANOVA also revealed a significant difference (F_1,22 _= 38.1; p < 0.0001) between groups (HFR vs. LFR); those at HFR took longer to completely cross the mat (average ± SEM was 81 ± 6 cm/s) than LFR older adults (129 ± 6 cm/s). There was a significant main effect of trials (F_3,66 _= 14.99; p < 0.0001). The average ± SEM Velocity was 102 ± 4 cm/s on trial 1, 105 ± 4 cm/s on trial 2, 108 ± 4 cm/s on trial 3, and 107 ± 4 cm/s on trial 4.

As with the previously discussed dependent measures, although the groups by lighting condition was not statistically significant, we performed post-hoc two-tailed paired t-tests comparing velocity for both groups under each lighting condition to verify if pathway plus night lights had a stronger impact on HFR than on LFR older adults. Older adults at HFR were significantly slower under night lights alone than under both ambient illumination (p < 0.0001) and pathway plus night lights (p = 0.010). Velocity under ambient illumination and under pathway plus night lights was not significantly different (p = 0.075). The average ± SEM Velocity was 86 ± 7 cm/s under ambient illumination, 76 ± 5 cm/s under night lights alone, and 81 ± 6 cm/s under pathway plus night lights. For the LFR participants, Velocity was significantly greater under ambient illumination than under both night lights alone (p = 0.0004) and pathway plus night lights (p < 0.0001), but there was no statistically significant difference in Velocity between night lights alone and pathway plus night lights (p = 0.159). The average ± SEM Velocity in LFR participants was 134 ± 7 cm/s under ambient illumination, 127 ± 5 cm/s under night lights alone, and 129 ± 6 cm/s under pathway plus night lights.

## Discussion

Consistent with reports in the literature [10], all of these older adults walked slower in dim light similar to that experienced by older adults when navigating in their bedrooms at night. This implies that reduced visibility causes older adults to move more slowly to minimize falls risk. As has been shown, higher risk of falls *is *associated with slower gait and lower scores on clinical balance scales [18-20]. Post hoc analyses of our data showed that the addition of laser lines to the dim light from night lights provided HFR participants with perceptual cues about the walking plane resulting in a significant increase in Velocity and a significant reduction in Step Length variability; therefore, dim light enhanced by visual perceptual cues was effective for improving postural stability and control in older adults with increased risk for falls. Although not as effective as bright ambient illumination, the addition of laser lines to the dim light from night lights resulted in a significantly reduced Stride Length variability in all participants (both HFR and LFR participants). The SD of the Stride Length Difference in LFR older adults was, however, greater than those at HFR; therefore, these results should be viewed with caution because LFR older adults were expected to have less variability in Stride Length than HFR older adults. In general, our results show that, compared to HFR older adults, LFR older adults were less impacted by the use of perceptual cues as measured by Step Length, SD of Step Length, and Velocity.

In addition to gait velocity, increased gait variability, which quantifies the stride fluctuations during walking, has also been associated with increased risk for falls in less active older adults [13,21]. It has been proposed that, in a between-subjects study, a 10 cm/sec drop in gait velocity is associated with a 7% higher risk for falls [18]. In another study, the same group of investigators proposed that a gait speed change in healthy older adults (age 70 years or older) of 4.15 cm/s was small, whereas a gait speed change of 10.38 cm/s was much more clinically significant [22]. In the present study, participants at HFR reduced their velocity from 85.6 cm/s to 75.6 cm/s, a change of 10 cm/s, when ambient lights were turned off and night lights were turned on. It is important to note that this drop in velocity occurred after 20 minutes of dark adaptation and it is expected that velocity would be reduced even more without dark adaptation. When the pathway plus night lights were added, the speed dropped to 81.5 cm/s, suggesting that the presence of perceptual cues about the horizontal walking plane was helpful in maintaining a walking speed closer to the ones observed under ambient lights. Whether this reduction in walking speed in a more challenging, dim environment can be related to increased risk of falls has yet to be investigated.

Brach et al. [22] proposed that a 0.25 cm change in Step Length SD is considered clinically meaningful. Although it was not clear from their report how SD was calculated, we compared the changes in Step Length SD in our study to determine whether there might be any clinical significance associated with our results. In the present study, Step Length SD was reduced by 0.99 cm for older adults at HFR when ambient illumination replaced the night lights, clearly a meaningful change. The pathway plus night lights increased Step Length SD by only 0.21 cm compared to ambient lights, which is not considered a clinically meaningful change according to Brach and colleagues. Again, using lighting to enhance perceptual cues about the horizontal walking plane may have a clinically significant, positive effect on reducing risk for falls, especially for those at higher falls risks.

Our observations from pilot studies, as well as the study by Itoh [12], revealed that older adults tend to look down when walking, especially in dim ambient light; therefore, a lighting system that provides perceptual cues about the walking plane may be *practically *significant as well. Of course, turning on bright ambient lights would be effective, but the high levels of illumination used in the present study are not common in homes during the evening and nighttime. In fact, bright lights at those times might not be an ideal scenario because sleep might be disrupted. Modest light levels could be as effective as bright lights, but if switches are not located near the bed or lights are not on motion sensors, older adults will be at a higher risk for falling if forced to navigate back to bed in the dim lighting afforded by night lights commonly used in the home.

Limitations of the study include a relatively small sample size. A larger sample size might have increased the statistical power so other measures of walking stability, such as Stride Length Difference, may have reached statistical significance. Generalizations of the present findings, which used healthy older adults, to others who have high-level gait disorders should only be done with caution. Nakamura et al. [23] showed that stride length variability in Alzheimer's disease patients was associated with increased risks for falls. Another application include obese children who exhibited shorter stride length and larger stride width while walking in a dark room illuminated with one night light compared to walking in a room with ambient lights [24]. The HFR older adults were significantly older than the LFR older adults (p = 0.04), and this age difference may have been a factor in the significant differences observed between groups. Because all subjects experienced all experimental conditions, we do not believe that the positive impact of the pathway lights observed in both groups was solely due to age. It would be interesting to repeat the experiment using an age-matched control group. Another limitation is the use of participants with normal visual acuity. Because the prevalence of visual impairment is significantly higher in older populations, particularly among older adults in nursing homes and assisted living facilities [25], the positive effects of lighting, including pathway lights, may be even more crucial for reducing falls. Although lighting to enhance perceptual cues may help increase postural control and stability in the two groups studied here, future empirical studies need to investigate the impact and efficacy of such lighting systems for other populations.

## Conclusions

The present results, together with those from Figueiro et al. [11] and Figueiro et al. [26] support the inference that lighting designed to provide veridical perceptual cues about the environment can help improve postural control and stability while, for example, older adults are changing from a sitting to a standing position and while they are walking. It is important to point out, however, that commercially available lighting systems similar to the pathway lights tested here are not available. This obviously limits the adoption of these simple, yet seemingly effective lighting solutions in the homes of older adults. It is hoped also that these lighting solutions will be seen by healthcare professionals as preventative measures to decrease falls. Architects and designers could then more readily implement lighting solutions to reduce falls in older adult living environments.

## Competing interests

The authors declare that they have no competing interests.

## Authors' contributions

MGF participated in the design of the experiment, data collection, data analyses, and wrote the manuscript. BP participated in data collection, analyses, and contributed to manuscript writing. MSR participated in data collection, contributed to data analyses, and reviewed final manuscript. LZG participated in data collection and reviewed final manuscript. MSR participated in the design of the experiment, data analyses, and manuscript writing. All authors read and approved the final manuscript.

## Pre-publication history

The pre-publication history for this paper can be accessed here:

http://www.biomedcentral.com/1471-2318/11/49/prepub
